# A Distribution-Independent Bound on the Level of Confidence in the Result of a Measurement

**DOI:** 10.6028/jres.102.040

**Published:** 1997

**Authors:** W. Tyler Estler

**Affiliations:** National Institute of Standards and Technology, Gaithersburg, MD 20899-0001

**Keywords:** level of confidence, measurement, probability, uncertainty

## Abstract

The Bienaymé-Chebyshev Inequality provides a quantitative bound on the level of confidence of a measurement with known combined standard uncertainty and assumed coverage factor. The result is independent of the detailed nature of the probability distribution that characterizes knowledge of the measurand.

## 1. Introduction

The ISO *Guide to the Expression of Uncertainty in Measurement* [1] and the NIST adaption [2] recommend that the result of a measurement of a quantity *Y* = *f*(*x*_1_, *x*_2_,⋯, *x_N_*) be reported as *Y* = *y* ± *U*, where *y* is the estimate (or expectation) of *Y* and *U* is an *expanded uncertainty* defined by *U* = *ku*_c_(*y*). Here *u*_c_(*y*) is the *combined standard uncertainty* and *k* is a *coverage factor* chosen to produce an interval having a level of confidence close to a desired value. For uncorrelated input quantities, the combined standard uncertainty is the positive square root of the variance

$$u_{c}^{2}(y)=\sum_{i=1}^{N}u_{i}^{2}(y),$$ (1)

where the terms $u_{i}^{2}(y)\equiv {\left(\partial f/\partial x_{i}\right)}^{2}u^{2}(x_{i})$ are weighted variances associated with the probability distributions that characterize one’s knowledge of the input quantities *x_i_*.

The level of confidence associated with a particular choice of coverage factor *k* is interpreted to mean that the true value of *Y* may be expected to lie in the interval *y ± ku*_c_(*y*) with an integrated, or cumulative, probability *P*(*k*). The *Guide* gives an extended discussion of the problem of establishing the relation between *k* and *P*, the details of which depend on the exact (and generally unknown) probability distribution associated with one’s knowledge of the measurand *Y*.

This note describes a very general and useful bounding relation, long known to professional statisticians, that is free of these details. An interesting collection of such relations was compiled and described by I. R. Savage [3]. Our motivation derives from the growing acceptance and use of the *Guide* in engineering metrology and industrial quality control, and a perceived need for a wider exposure to some of the fundamental ideas of probability theory.

## 2. The Bienaymé-Chebyshev Inequality

This simple but general result was first derived by the French mathematician I. J. Bienaymé (1853) and rediscovered by P. L. Chebyshev (1867). We follow the development of Kenney and Keeping [4]. Consider a general probability density function *p*(*Y*) that satisfies the basic requirements of being normalized and having finite mean *y* and variance σ^2^:

$$\begin{array}{c} 1=\int_{-\infty }^{+\infty }p(Y)dY\\ y=\int_{-\infty }^{+\infty }Yp(Y)dY\\ \sigma^{2}=\int_{-\infty }^{+\infty }{\left(Y-y\right)}^{2}p(Y)dY. \end{array}$$ (2)

Now let *a* be an arbitrary positive constant. The probability that |*Y* – *y*| ≥ *a* is given by:

$$Pr(|Y-y|\geq a)=\int_{R}p(Y)dY,$$ (3)

where *R* denotes the set of values of *Y* that satisfy the stated inequality. Now clearly

$$\sigma^{2}\geq \int_{R}{\left(Y-y\right)}^{2}p(Y)dY\geq a^{2}\int_{R}p(Y)dY,$$ (4)

so that from Eqs. (3) and (4) we have immediately:

$$Pr(|Y-y|\geq a)\leq \frac{\sigma^{2}}{a^{2}}.$$ (5)

This result is known as the Bienaymé-Chebyshev Inequality. Setting *a* = *kσ*, Eq. (5) becomes:

$$Pr(|Y-y|\geq k\sigma )\leq \frac{1}{k^{2}}.$$ (6)

We thus see that *independent of the detailed nature of the distribution p*(*Y*), the probability that the true value of *Y* will differ from its expected (or measured) value *y* by as much as *k* standard deviations is not more than 1/*k*^2^. Now for *k* ≤ 1, this is not very informative, but for larger values of *k* it becomes quite interesting. *It tells us, for example, that not less than* 8/9 ≈ 89 % *of the probability associated with a measurement of Y is contained in the interval y ±* 3*σ, whatever the distribution p*(*Y*). For a probability distribution known to be normal (or Gaussian), the corresponding result would be 99.7 %. While this is a significant improvement in the level of confidence, the lower value may be completely adequate for the measurement task at hand, while avoiding the need for nit-picking over the details of the exact form of the distribution *p*(*Y*).
